# Clinically validated immune-related gene markers and molecular subtypes in acute myocardial infarction revealed by peripheral blood transcriptomics

**DOI:** 10.3389/fcvm.2026.1643959

**Published:** 2026-01-23

**Authors:** Qingquan Zhang, Mingyan Yu, Peiran Xu, Louyuan Xu, Zhe Wang, Liang Chen, Koulong Zheng

**Affiliations:** Department of Cardiology, The Second Affiliated Hospital of Nantong University, Nantong First People’s Hospital, Nantong, Jiangsu, China

**Keywords:** acute myocardial infarction, bioinformatics analysis, immune infiltration, molecular subtype, WGCNA

## Abstract

**Background:**

Acute myocardial infarction (AMI) is a leading cause of morbidity and mortality worldwide. Beyond ischemic injury, sterile inflammation and immune activation critically shape infarct expansion, healing, and adverse remodeling. However, immune-related genes (IRGs) that distinguish AMI from stable coronary artery disease (sCAD) and reflect patient heterogeneity remain incompletely characterized.

**Methods:**

Two microarray datasets (GSE59867 and GSE62646) were retrieved from database and integrated after batch correction. Differential expression analysis and weighted gene co-expression network analysis (WGCNA) were combined with CIBERSORT to identify differentially expressed immune-related genes (DEIRGs) and hub genes associated with immune infiltration. Consensus clustering was then applied to explore molecular subtypes of AMI. Finally, hub genes were preliminarily validated by RT-qPCR in a clinical cohort and in an independent public dataset (GSE60993).

**Results:**

A total of 155 differentially expressed genes (DEGs) and 27 DEIRGs were identified. WGCNA highlighted the MEblue module as most strongly associated with AMI, and intersection analysis yielded 13 overlapping DEIRGs. Protein-protein interaction analysis prioritized six hub genes (CSF3R, CD14, AQP9, S100A9, SLC11A1, and IL1RN), which were mainly correlated with neutrophil and monocyte fractions. Consensus clustering indicated three molecular subtypes with distinct hub-gene expression patterns. RT-qPCR confirmed significantly increased expression of AQP9, S100A9, and SLC11A1 in AMI compared with sCAD. External validation in GSE60993 supported the diagnostic potential of the identified genes.

**Conclusions:**

AQP9, S100A9, and SLC11A1 are promising immune-related biomarkers and may reflect heterogeneity in inflammatory responses among AMI patients. These findings provide mechanistic clues and candidate targets for future experimental and translational studies.

## 1 Introduction

Acute myocardial infarction (AMI) remains a major cause of cardiovascular death despite advances in reperfusion and secondary prevention (1). AMI typically occurs on the background of coronary atherosclerosis and is frequently triggered by plaque disruption, thrombosis, and abrupt coronary occlusion. Although cardiac troponins are central to diagnosis, there is a continuing need to better understand the molecular mechanisms that drive the transition from stable coronary artery disease (sCAD) (2) to acute coronary events and to identify biomarkers that reflect immuno-inflammatory activity.

Chest pain is the earliest and most common clinical manifestation of AMI. Diagnosis is typically based on characteristic clinical symptoms, elevated levels of cardiac troponin I (cTnI), and electrocardiographic (ECG) changes, such as ST-T abnormalities. However, cTnI elevation often takes 3–4 h to become detectable. While myoglobin levels rise more rapidly, they are influenced by various non-cardiac factors, making myoglobin a less specific biomarker and more suitable as an auxiliary diagnostic indicator. Moreover, in NSTEMI cases, ECG often fails to show significant ST-segment elevation. Since AMI itself triggers a local and systemic inflammatory response (3–5), identifying novel biomarkers capable of detecting this immune-inflammatory reaction at an earlier stage could potentially improve the early prediction and diagnosis of AMI, particularly in patients with sCAD, before it progresses to a full-blown infarction. Such markers could also enable more precise and timely therapeutic interventions in the early stages of AMI. Sterile inflammation is initiated immediately after myocardial ischemia through the release of damage-associated molecular patterns (DAMPs) that activate pattern-recognition receptors and downstream pathways such as TLR/NF-kappaB signaling, inflammasome activation, and cytokine cascades (6, 7). Neutrophils and monocytes are rapidly mobilized and recruited to the infarcted myocardium, where they contribute to tissue injury in the early phase but also participate in debris clearance and subsequent repair. Clinically and experimentally, the balance and timing of these immune responses are strongly linked to infarct size, healing quality, ventricular remodeling, and heart failure risk (8).

Importantly, AMI is not immunologically homogeneous. Transcriptomic and single-cell studies have suggested that circulating immune cell subsets and inflammatory gene programs vary across patients and may correlate with plaque phenotypes and clinical outcomes (9). Therefore, integrating immune infiltration estimation with gene co-expression and clustering analyses may help identify immune-related genes and molecular subtypes relevant to AMI progression and patient stratification. In this study, we integrated two microarray datasets to identify immune-related differentially expressed genes and construct an AMI-associated co-expression network. We combined differential expression analysis, WGCNA, immune cell deconvolution, protein-protein interaction (PPI) analysis, and consensus clustering to determine hub immune genes and molecular subtypes. Finally, we performed preliminary validation by RT-qPCR in a clinical cohort and by re-analysis of an independent dataset.

## 2 Materials and methods

### 2.1 Bioinformatics workflow

#### 2.1.1 Data downloading and preprocessing

Gene expression profiles of PBMCs from patients with ST-segment elevation myocardial infarction (STEMI) and stable coronary artery disease (sCAD) were downloaded from the Gene Expression Omnibus (GEO) database (10). Two datasets [GSE59867 (11) and GSE62646 (12)] generated on the GPL6244 Affymetrix Human Gene 1.0 ST Array platform were included. GSE59867 contained PBMC samples from 111 STEMI patients and 46 patients with stable CAD without prior myocardial infarction; GSE62646 contained PBMC samples from 28 STEMI patients and 14 stable CAD patients. Raw data were background-corrected and normalized, and the two datasets were merged. Batch effects were removed using the ComBat method implemented in the sva package (13).

#### 2.1.2 Identification of differentially expressed genes

Differential expression analysis between AMI (STEMI) and sCAD samples was performed using the limma package (14). Genes with adjusted *P* value <0.05 and |log2 fold change| > 0.5 were considered differentially expressed genes (DEGs). Volcano plots and heatmaps were generated to visualize DEGs (15). Gene set enrichment analysis (GSEA) and functional enrichment analyses were conducted using clusterProfiler package (16).

#### 2.1.3 Identification and functional annotation of immune-related DEGs

A curated list of immune-related genes (IRGs) was downloaded from the ImmPort database (17). Immune-related differentially expressed genes (DEIRGs) were identified by intersecting DEGs with the IRG list. Gene Ontology (GO) and Kyoto Encyclopedia of Genes and Genomes (KEGG) pathway enrichment analyses were performed for DEIRGs using clusterProfiler package. Adjusted *P* value <0.05 was considered statistically significant.

#### 2.1.4 Weighted gene co-expression network analysis

Weighted gene co-expression network analysis (WGCNA) (18) was conducted to identify co-expression modules related to AMI status. An appropriate soft-thresholding power was selected to construct a scale-free topology network. Modules were identified by hierarchical clustering and dynamic tree cutting, and module-trait relationships were assessed. The module most strongly associated with AMI was selected for downstream analysis.

#### 2.1.5 Screening of Hub genes

Overlapping genes between the key WGCNA module and DEIRGs were obtained. Protein-protein interaction (PPI) analysis of overlapping genes was performed using the STRING database and visualized in Cytoscape. Hub genes were prioritized based on network connectivity and biological relevance.

#### 2.1.6 Immune cell infiltration analysis

CIBERSORT was used to estimate the relative proportions of 22 immune cell types from the normalized gene expression matrix using linear support vector regression (19). Only samples with CIBERSORT output *P* < 0.05 were retained for downstream immune-infiltration analyses. Correlation among immune cell fractions and differences between AMI and sCAD groups were visualized. Spearman correlation analysis was performed to assess relationships between hub gene expression and immune cell fractions (20).

#### 2.1.7 Identification of molecular subtypes in AMI

ConsensusClusterPlus (21) package were used to perform unsupervised consensus clustering of AMI samples based on the expression of overlapping DEIRGs. The optimal cluster number (K) was determined using consensus cumulative distribution function (CDF) curves and consensus matrices. Differences in hub gene expression among molecular subtypes were evaluated.

### 2.2 Clinical cohort and RT-qPCR validation

After excluding relevant contraindications, peripheral blood samples were collected from 10 clinically confirmed AMI patients and 10 patients with stable CAD at the Second Affiliated Hospital of Nantong University. PBMCs were isolated, and total RNA was extracted for reverse transcription. Real-time quantitative PCR was performed on an Applied Biosystems 7500 system. Each 20 uL reaction contained 2 uL cDNA, 10 uL PCR master mix, 0.4 uL of each primer (10 uM, sequences shown in Table 1), and nuclease-free water. Cycling conditions were 95C for 2 min, followed by 40 cycles of 95 °C for 15 s and 60 °C for 30 s. GAPDH served as the internal control, and relative expression levels were calculated using the 2^−ΔΔCt^ method.

**Table 1:** Primer sequences.

Gene	Oligo name	Sequence (5’ → 3’)
AQP9	FORWARD	TGGCGGTGTCTCTGGTGGTC
	REVERSE	CCCCACAAAGGCTCCCAAGAAC
S100A9	FORWARD	CACGGCCACAGCCACTAATCAG
	REVERSE	CCCCTAGCCCCACAGCCAAG
SLC11A1	FORWARD	GCCGAGCAGACATCAGAGAAGC
	REVERSE	TGTTGAACGCAGCCTGGTTGG
IL1RN	FORWARD	GCTTCGCCTTCATCCGCTCAG
	REVERSE	GCTTCCATCGCTGTGCAGAGG
CD14	FORWARD	ACTGATGGCGGCTCTCTGTCC
	REVERSE	TGTGGCTGAGGTCTAGGCTGTG
CSF3R	FORWARD	GCTGTTGCTCACCTGCCTCTG
	REVERSE	AGCTGGGTCTGGGACACTTGG
GAPDH	FORWARD	GGTTGTCTCCTGCGACTTCA
	REVERSE	TGGTCCAGGGTTTCTTACTCC

### 2.3 External validation dataset and ROC analysis

To complement the lack of healthy controls in the clinical validation cohort, the publicly available dataset GSE60993 was used as an independent validation set. This dataset includes whole blood gene expression profiles from patients with acute coronary syndrome and healthy controls. We re-analyzed the subset of STEMI/AMI samples and healthy controls to validate the expression patterns of hub genes. Receiver operating characteristic (ROC) curves were generated to evaluate the discriminatory performance of candidate genes.

### 2.4 Statistical analysis

Bioinformatics analyses were conducted in R (version 4.1.2). Clinical data and RT-qPCR results were analyzed using IBM SPSS Statistics 26.0 and GraphPad Prism 9.0. For comparisons between two groups, Student's *t*-test was used for normally distributed data and the Mann–Whitney *U* test was used for non-normally distributed data. *P* < 0.05 was considered statistically significant.

## 3 Results

### 3.1 Data preprocessing

**Figure 1:**
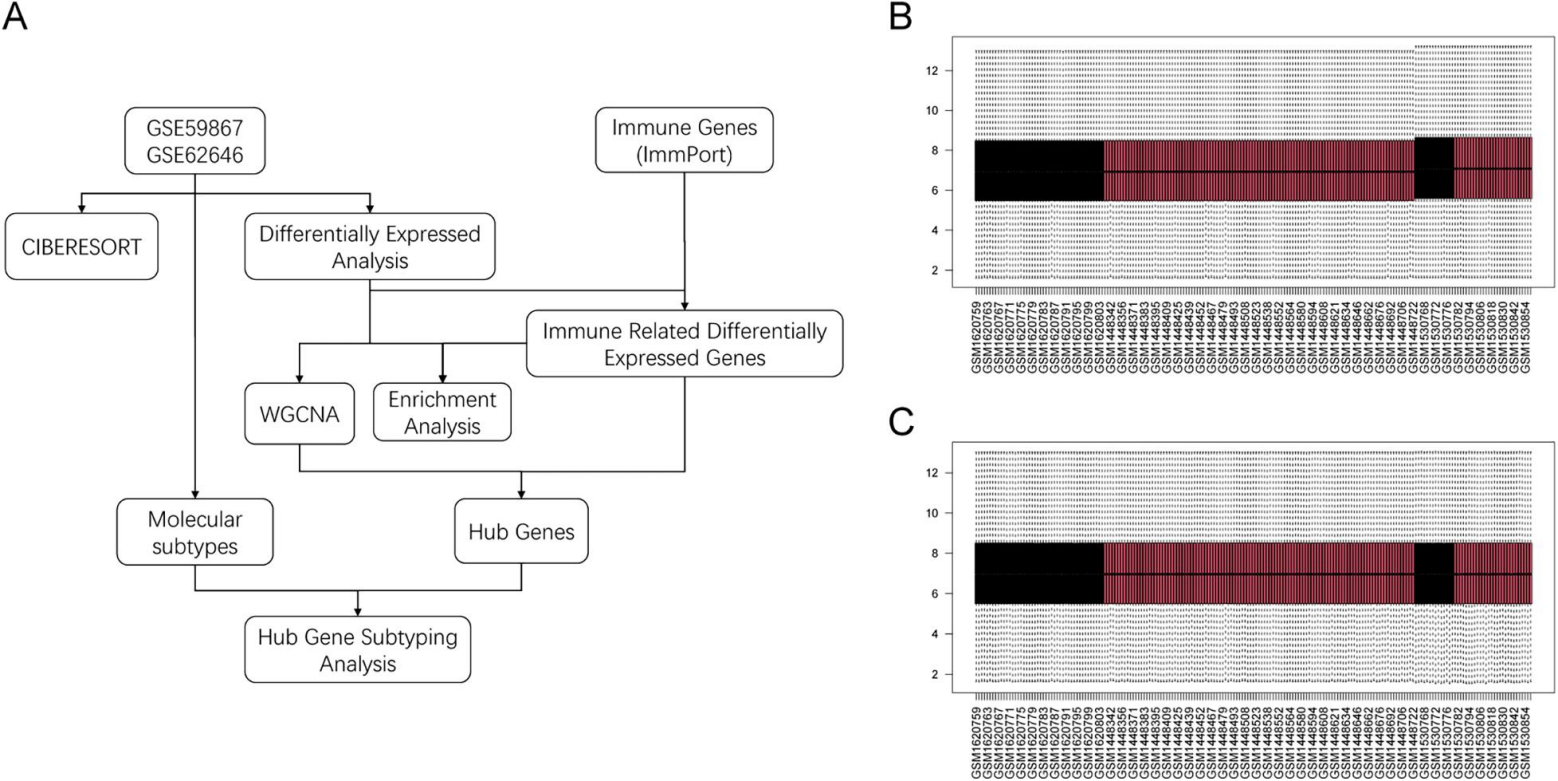
Study workflow and batch-effect correction. **(A)** Overall workflow of the study. **(B)** Boxplot of merged datasets before batch correction. **(C)** Boxplot after ComBat batch correction.

The flowchart of this study is shown in Figure 1A. The gene expression matrices of GSE59867 and GSE62646 were normalized and merged, followed by batch-effect removal. Boxplots before and after batch correction indicated improved comparability across samples (Figures 1B,C).

### 3.2 Differential analysis of infiltrated immune cells

**Figure 2:**
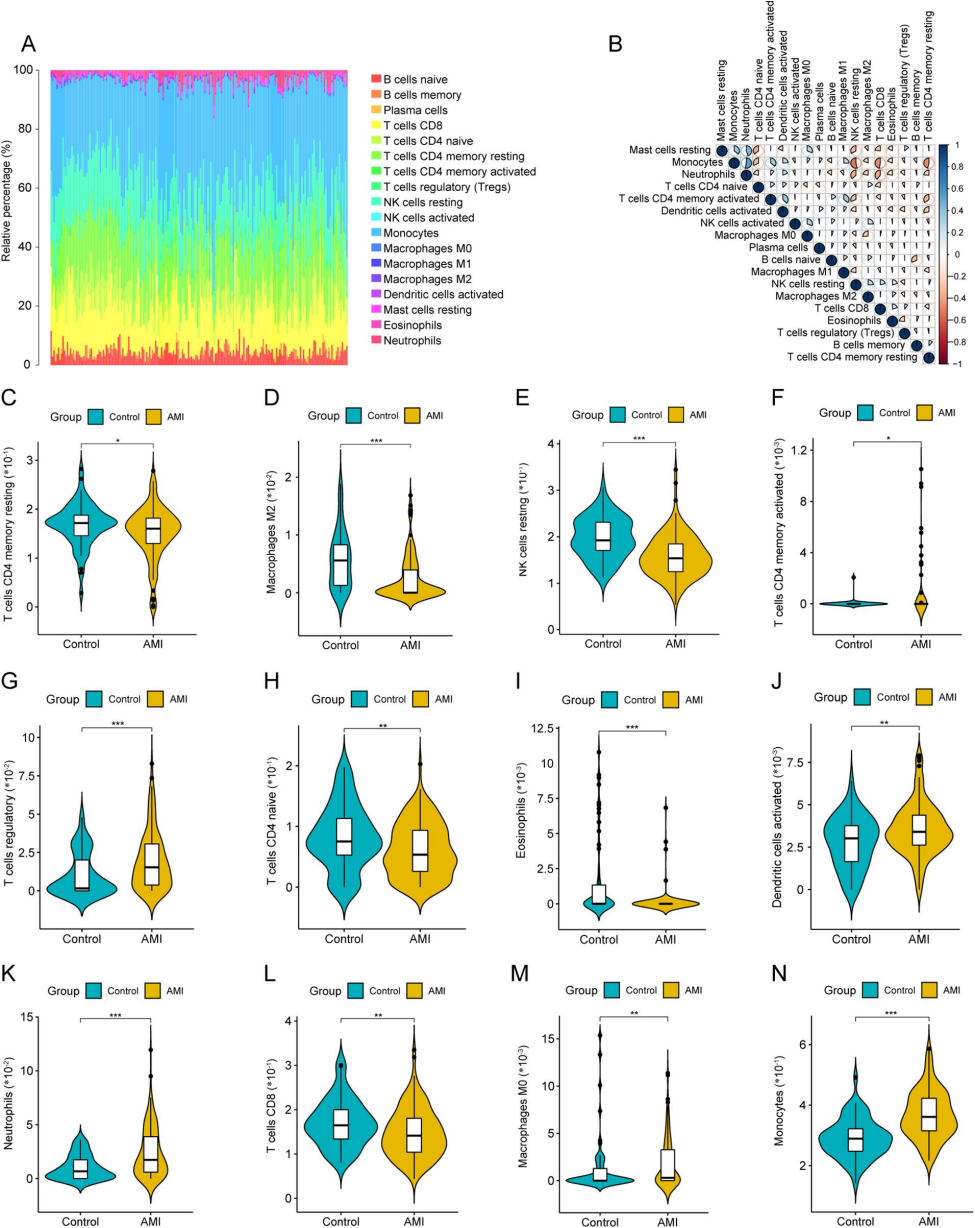
Immune cell infiltration analysis by CIBERSORT. **(A)** Stacked barplot showing relative proportions of 22 immune cell types in each sample. **(B)** Correlation heatmap among immune cell fractions. **(C–N)** Violin plots comparing immune cell fractions between AMI and sCAD groups (CIBERSORT *P* < 0.05).

The relative proportions of 22 immune cell types differed across samples (Figure 2A). Correlation analysis suggested coordinated variation among several immune populations (Figure 2B). Compared with sCAD, AMI samples showed significant differences in multiple immune cell subsets, including resting memory CD4T cells, M2 macrophages, and resting NK cells (Figures 2C–N).

### 3.3 Identification and enrichment analysis of DEGs

**Figure 3:**
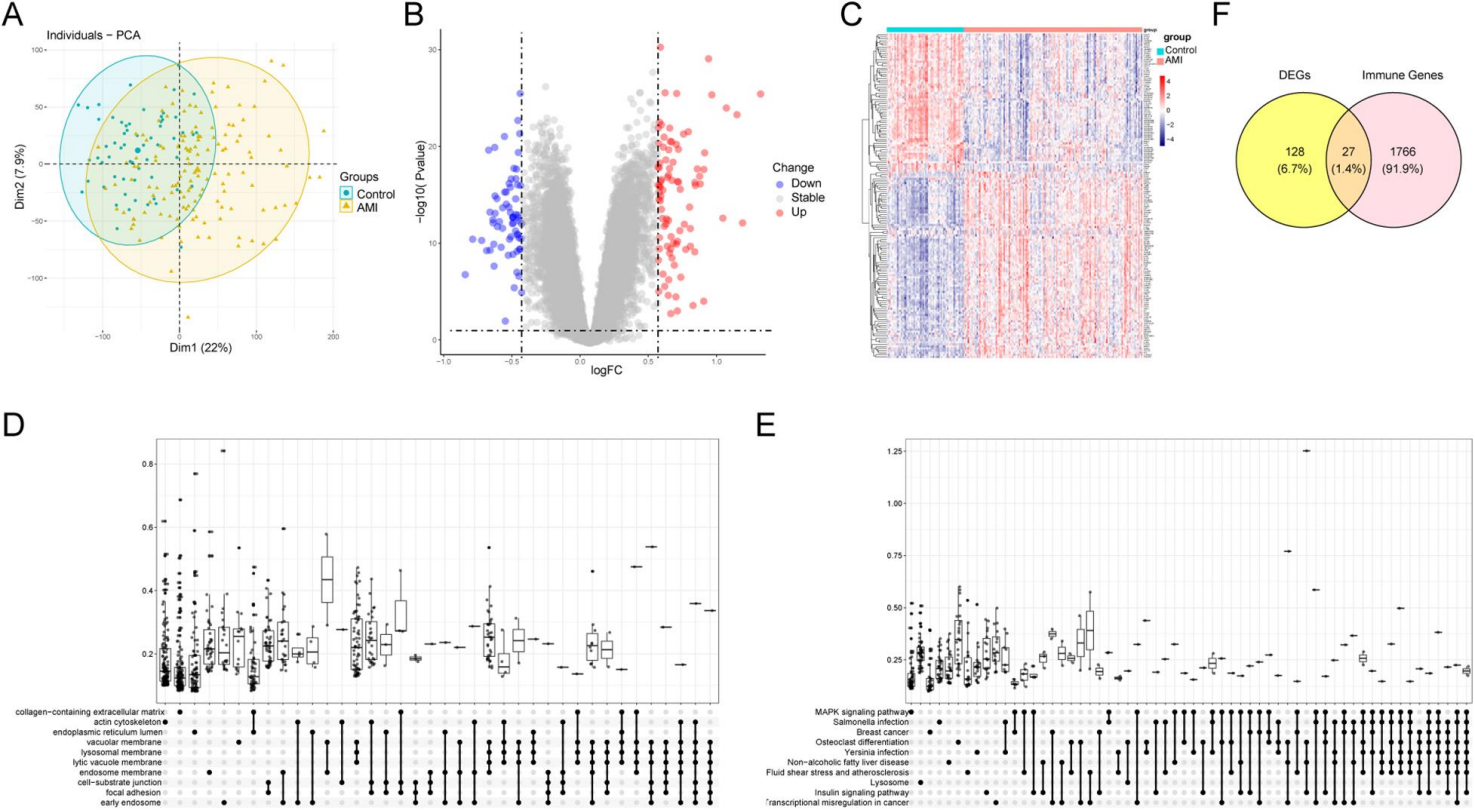
Differential expression analysis and enrichment. **(A)** PCA plot of merged datasets. **(B)** Volcano plot of DEGs (adj.*P* < 0.05, |log2FC| > 0.5). **(C)** Heatmap of representative DEGs. **(D,E)** GO and KEGG enrichment analyses of DEGs. **(F)** Venn diagram showing DEIRGs.

Principal component analysis (PCA) indicated separation between AMI and sCAD samples based on global gene expression (Figure 3A). Differential expression analysis identified 155 DEGs (Figures 3B,C). GO and KEGG enrichment analyses suggested that DEGs were enriched in immune- and inflammation-related biological processes and pathways (Figures 3D,E; Supplementary Tables S1 and S2). Intersecting DEGs with the ImmPort IRG list yielded 27 DEIRGs (Figure 3F).

### 3.4 Functional analysis of DEIRGs

**Figure 4:**
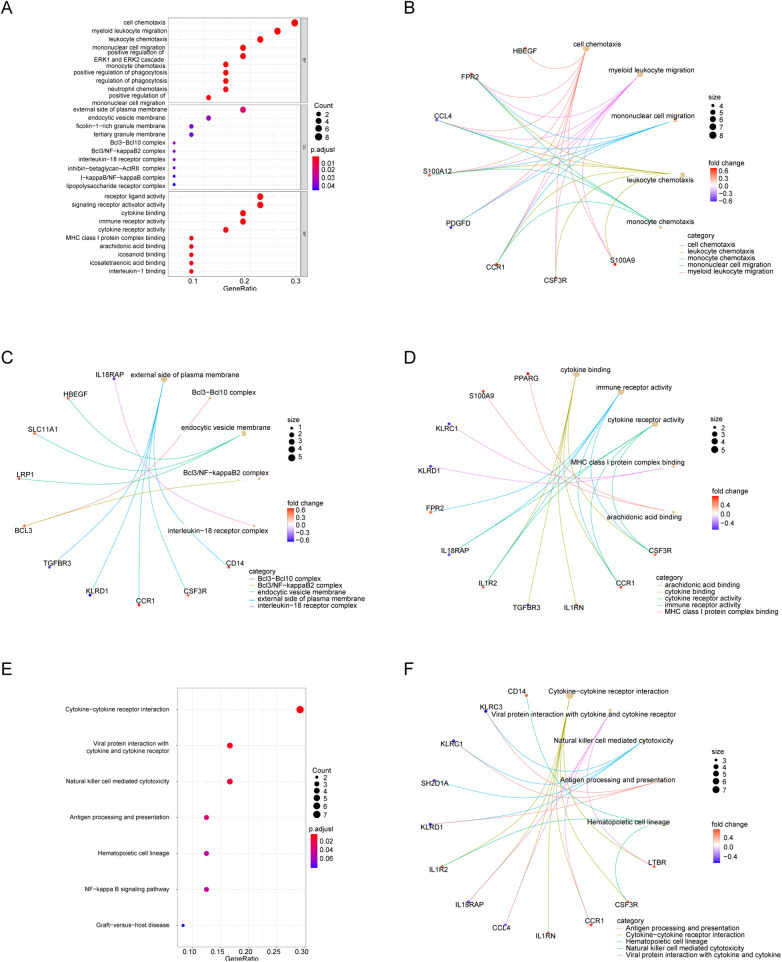
Functional enrichment of DEIRGs. **(A)** GO bubble plot (BP). **(B–D)** GO networks for BP, CC, and MF. **(E)** KEGG bubble plot. **(F)** KEGG network.

GO and KEGG enrichment analyses of DEIRGs were performed to characterize immune-related functional signatures. GO terms were mainly associated with cell chemotaxis, myeloid leukocyte migration, and mononuclear cell migration. KEGG pathways were enriched in cytokine-cytokine receptor interaction, viral protein interaction with cytokines and cytokine receptors, and NK cell-mediated cytotoxicity (Figure 4; Supplementary Tables S3 and S4).

### 3.5 WGCNA analysis

**Figure 5:**
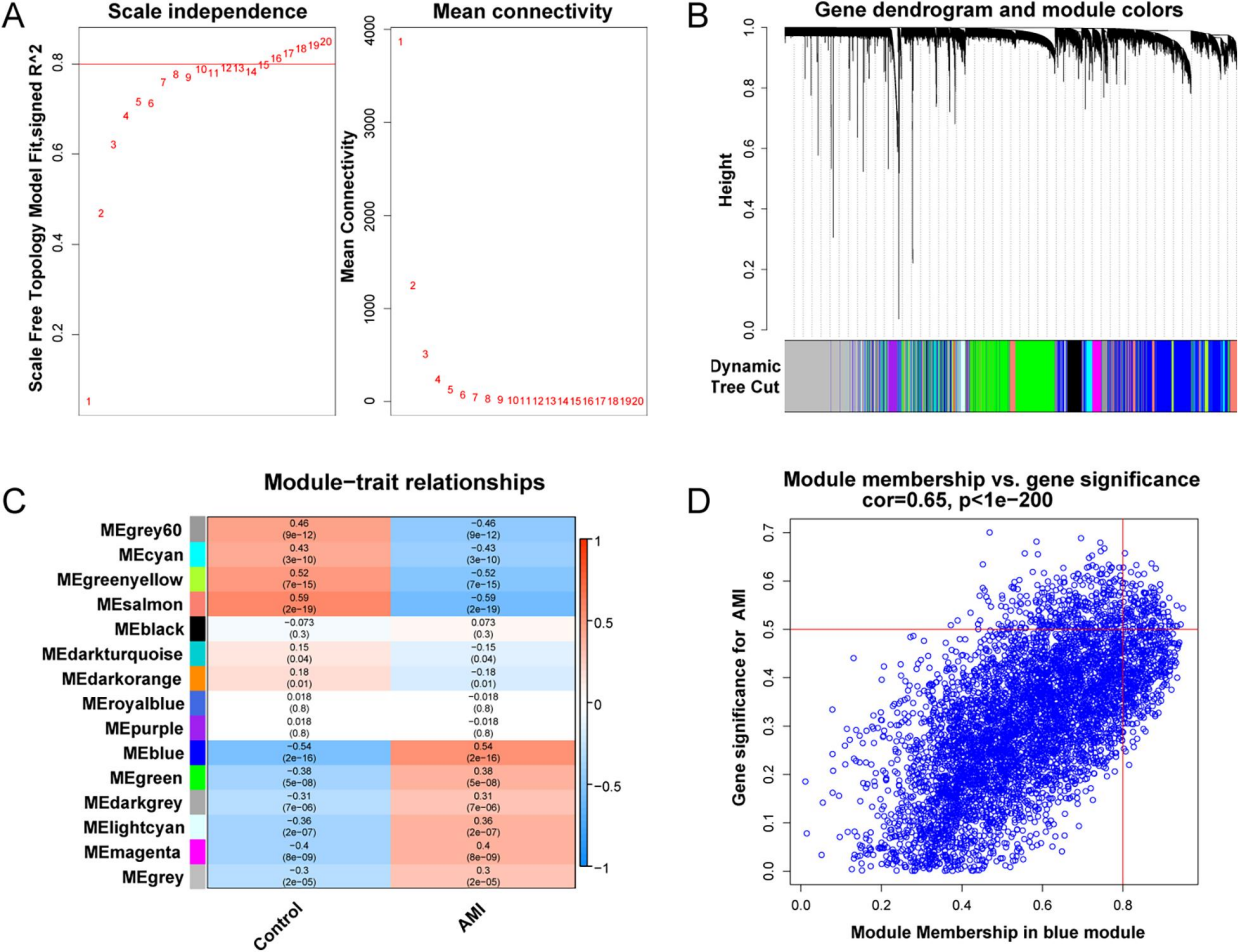
WGCNA analysis. **(A)** Soft-threshold power selection. **(B)** Gene dendrogram and module colors. **(C)** Module-trait relationship heatmap. **(D)** Scatter plot of gene significance vs. module membership for MEblue.

WGCNA was applied to identify co-expression modules associated with AMI. Sample clustering did not reveal major outliers among 199 samples. A soft-thresholding power of 10 was selected to approximate a scale-free topology (Figure 5A). Among the identified modules, the blue module (MEblue) showed the strongest association with AMI status (*P* = 2 × 10^−16^; Figures 5B–D) and was selected for downstream analyses.

### 3.6 Hub genes and correlation with immune infiltration

**Figure 6:**
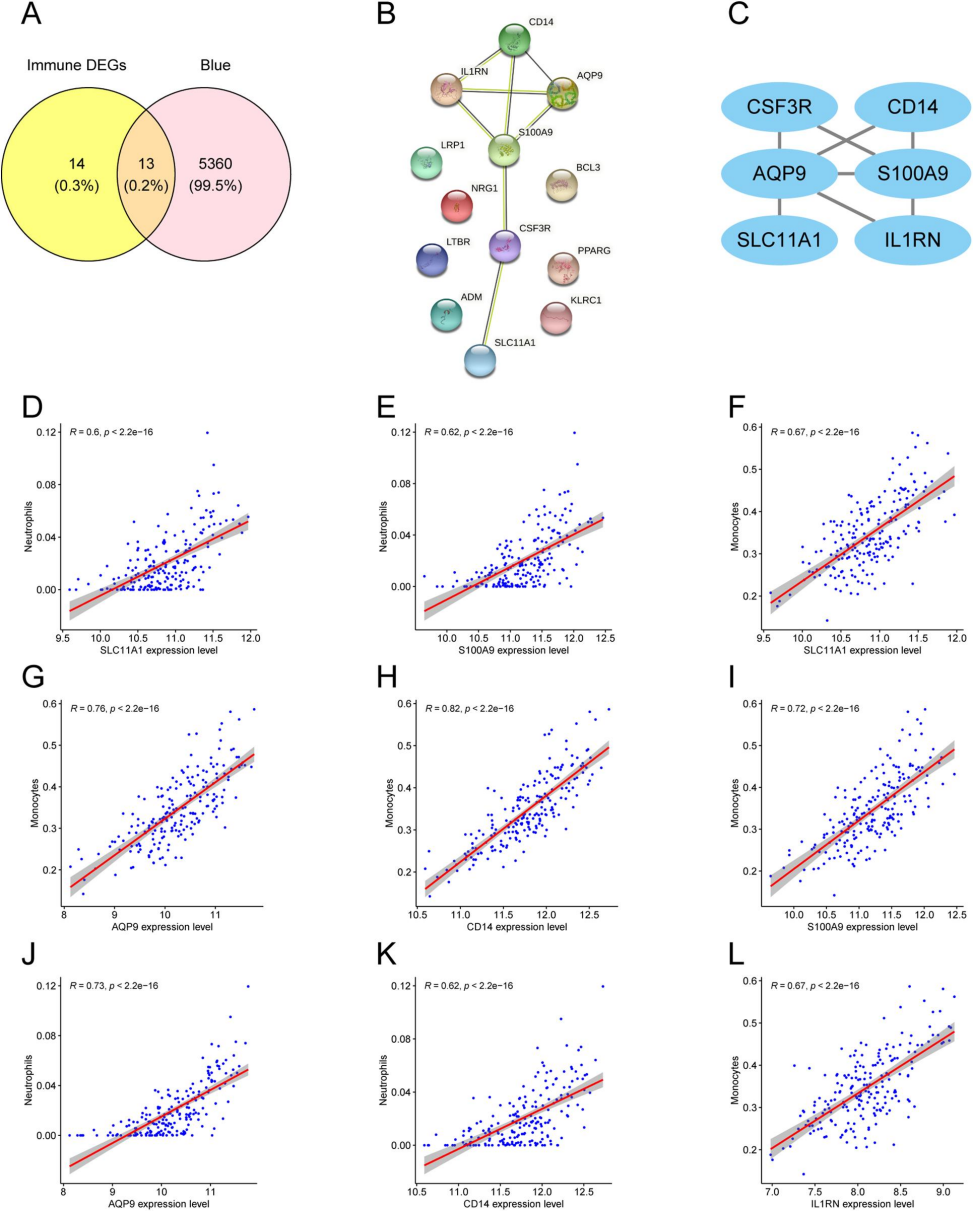
Hub gene screening and immune correlation. **(A)** Venn diagram of DEIRGs and MEblue module genes. **(B)** PPI network of 13 overlapping DEIRGs. **(C)** Highlighted hub genes within the PPI network. **(D–L)** Scatter plots showing correlations between hub genes and immune cell fractions.

To connect the AMI-associated MEblue module with immune dysregulation, DEIRGs were intersected with MEblue module genes, yielding 13 overlapping DEIRGs (Figure 6A). PPI analysis of these genes identified six hub genes: CSF3R, CD14, AQP9, S100A9, SLC11A1, and IL1RN (Figures 6B,C). Correlation analysis (*R* ≥ 0.6, *P* < 0.05) indicated that hub gene expression was primarily associated with neutrophil and monocyte fractions (Figures 6D–L).

### 3.7 Identification of molecular subtypes in AMI

**Figure 7:**
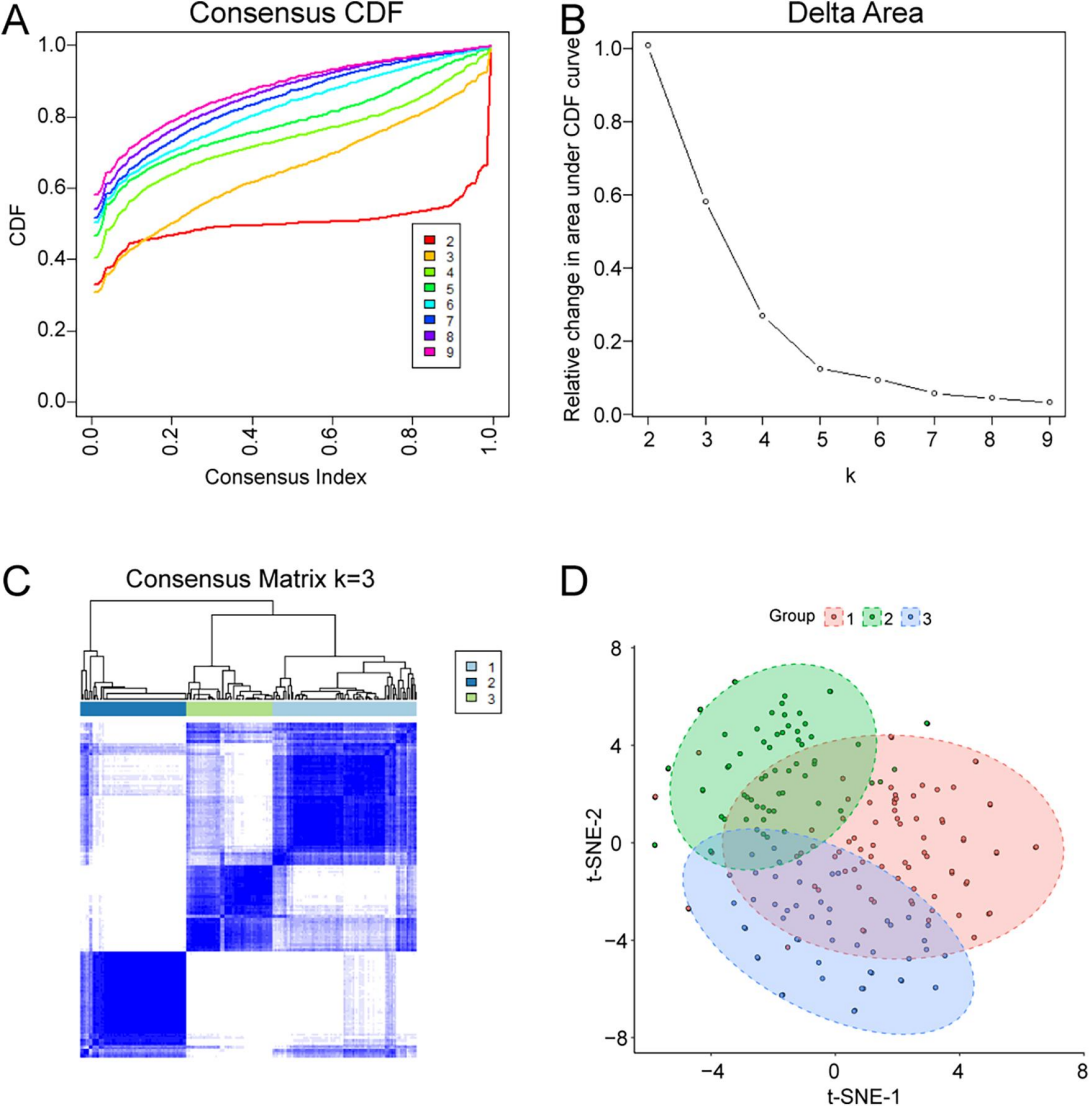
Identification of molecular subtypes in AMI by consensus clustering. **(A)** Consensus CDF curves for different *K* values. **(B)** Relative change in area under the CDF curve. **(C)** Consensus matrix heatmap for *K* = 3. **(D)** Cluster assignment and sample distribution visualization.

Consensus clustering was performed to explore molecular heterogeneity among AMI patients. Based on the CDF curve and consensus matrices, *K* = 3 provided stable clustering with minimal inter-cluster ambiguity (Figures 7A–C). Accordingly, AMI samples were divided into three clusters: Cluster 1 (*n* = 85), Cluster 2 (*n* = 63), and Cluster 3 (*n* = 51). The distribution of the three clusters is shown in Figure 7D.

### 3.8 Hub gene expression across molecular subtypes

**Figure 8:**
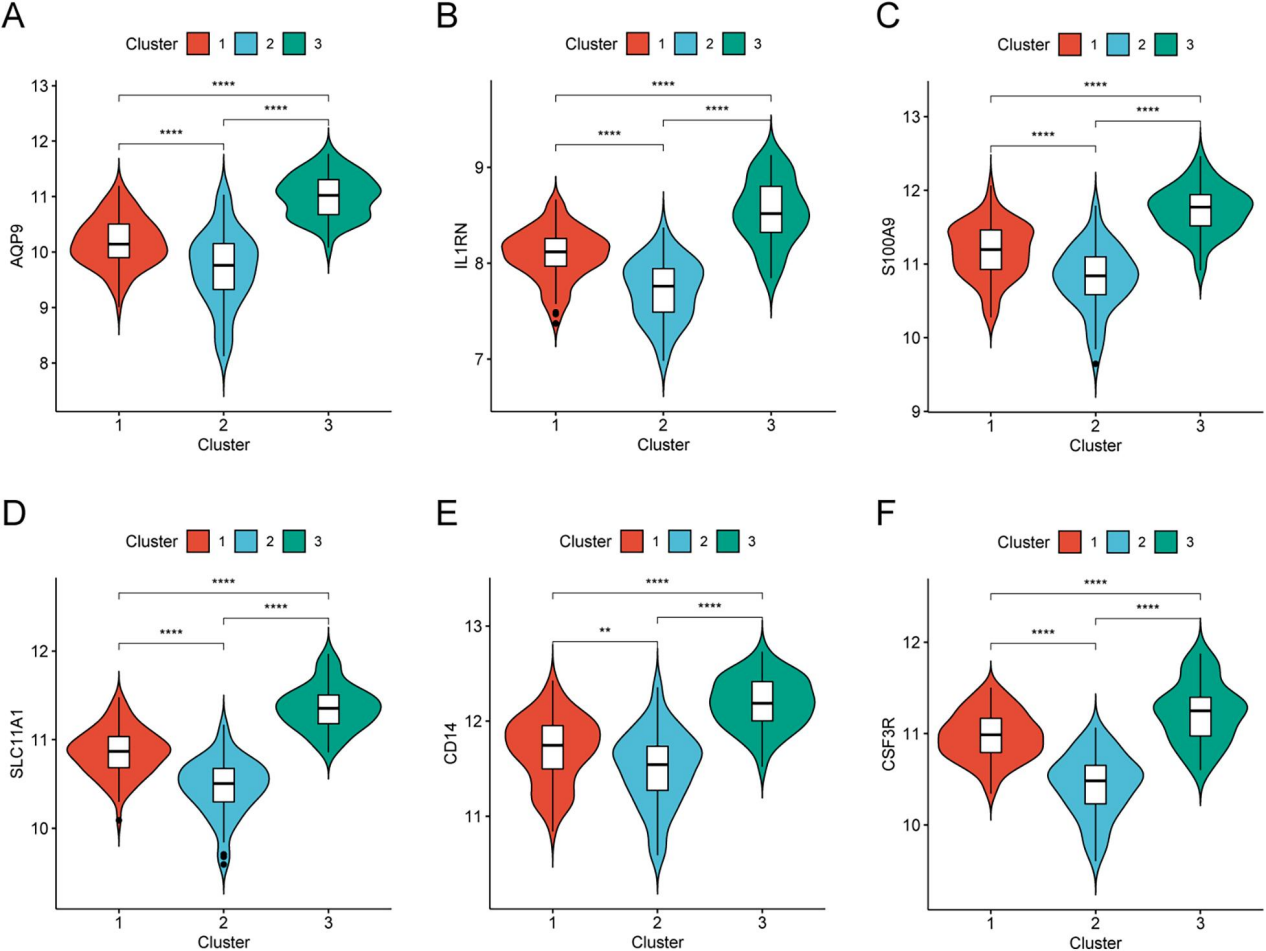
Hub gene expression in AMI molecular subtypes. **(A–F)** Expression levels of CSF3R, CD14, AQP9, S100A9, SLC11A1, and IL1RN across the three clusters.

To further characterize the clusters, the expression patterns of the six hub genes were compared among Cluster 1, Cluster 2, and Cluster 3. All six hub genes showed the highest expression in Cluster 3 and the lowest expression in Cluster 2 (Figures 8A–F), suggesting that Cluster 3 may represent a higher inflammatory/innate immune activation subtype.

### 3.9 Validation of hub genes in the clinical cohort and external dataset

**Figure 9:**
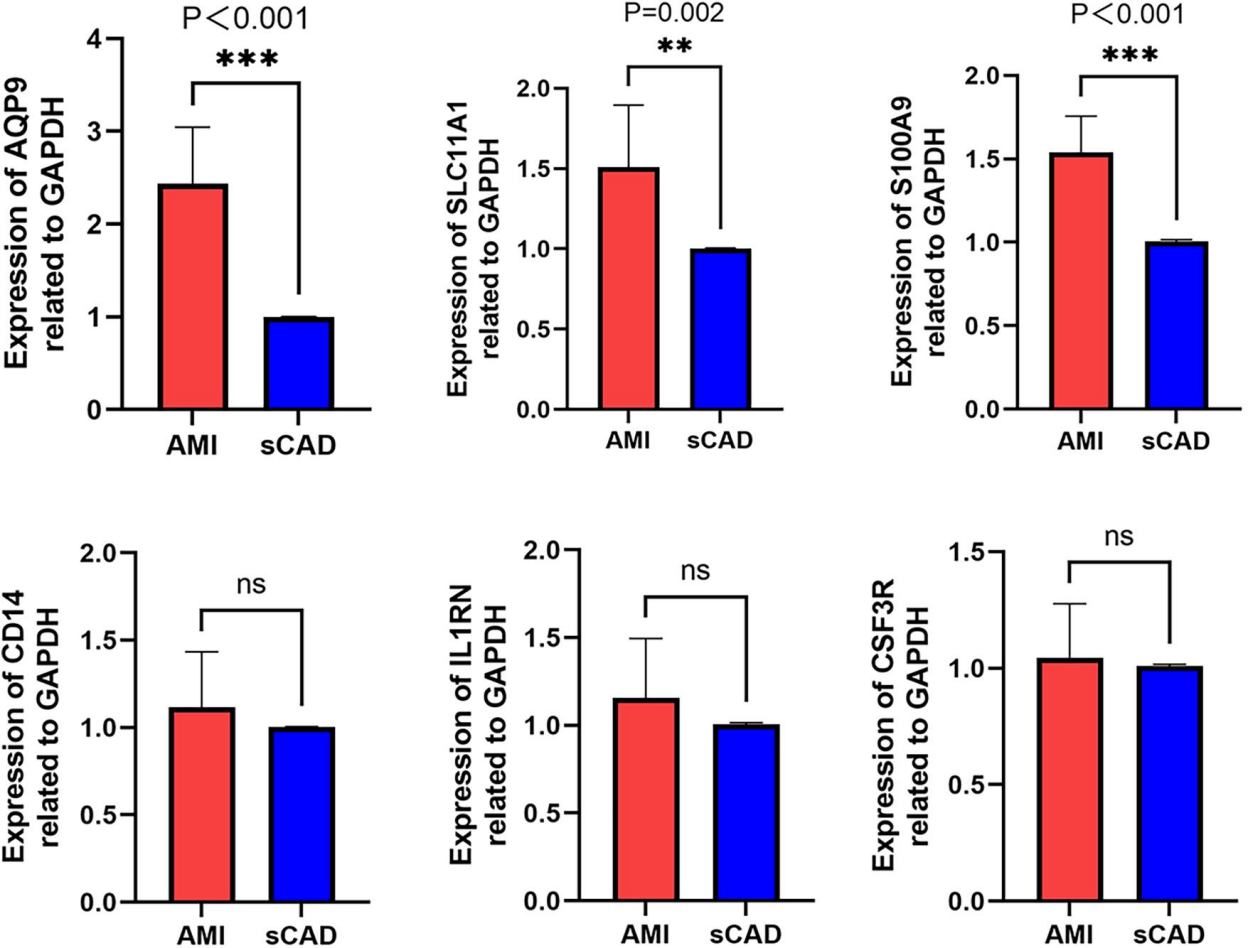
RT-qPCR validation of hub genes. Relative mRNA expression levels of CSF3R, CD14, AQP9, S100A9, SLC11A1, and IL1RN in PBMCs from AMI patients (*n* = 10) and sCAD patients (*n* = 10).

RT-qPCR was performed in PBMCs from 10 AMI patients and 10 sCAD patients. AQP9 and S100A9 were significantly upregulated in AMI, and SLC11A1 also showed higher expression in AMI than in sCAD. No significant differences were observed for CSF3R, CD14, or IL1RN in this small cohort (Figure 9). To address the absence of healthy controls in our clinical cohort, we re-analyzed the public dataset GSE60993 as an independent validation cohort. The identified genes were upregulated in AMI/STEMI samples compared with healthy controls, and ROC analysis suggested discriminatory ability for AMI (Supplementary Figure S1).

## 4 Discussion

In this study, we integrated PBMC transcriptomic datasets to identify immune-related genes and molecular subtypes associated with AMI. By combining differential expression analysis, WGCNA, immune cell deconvolution, and PPI network analysis, we identified six hub immune genes (CSF3R, CD14, AQP9, S100A9, SLC11A1, and IL1RN) that were strongly linked to neutrophil and monocyte fractions. Our enrichment analyses highlighted leukocyte chemotaxis/migration and cytokine signaling, consistent with the established concept that AMI triggers rapid systemic and local immune activation. Neutrophils and monocytes are among the earliest responders and can amplify injury through proteases, reactive oxygen species, and inflammatory cytokines, while later reparative programs facilitate resolution and scar formation. Therefore, circulating immune signatures may capture both disease activity and heterogeneity in inflammatory states among AMI patients.

The molecular subtypes identified by consensus clustering provide an additional perspective on AMI heterogeneity. Cluster 3 exhibited consistently higher hub gene expression, suggesting a subgroup with more pronounced innate immune activation, whereas Cluster 2 showed the lowest expression. Future studies integrating clinical outcomes, imaging features, and longitudinal sampling are needed to determine whether these transcriptomic subtypes correspond to differences in plaque phenotype, reperfusion injury, infarct healing, or prognosis.

We then conducted a clinical data analysis of the six selected genes. First, we enrolled clinically confirmed AMI patients and matched them with sCAD controls. The results showed that three genes—AQP9, S100A9 and SLC11A1—were significantly upregulated in AMI patients, suggesting that they may be involved in the pathophysiological processes of myocardial infarction. However, the expression levels of CSF3R, IL1RN and CD14 showed no significant difference between the AMI and sCAD groups, which was not entirely consistent with our previous bioinformatics predictions. We speculate that there might be the following reasons: ① Differences in data acquisition methods and methodological distinctions in detection approaches have led to inconsistencies between bioinformatics analysis data and validation cohort. This discrepancy may be attributed to the standardized processing of high-throughput sequencing data, whereas RT-qPCR relies on amplification signals from specific primers, potentially leading to deviations in the expression trends of certain genes between the two methods.② It is undeniable that our sample size is relatively small, which may cause errors in the research results. ③ Furthermore, since RT-qPCR typically analyzes specific samples while high-throughput data may encompass multiple patients, physiological variations among individuals could also contribute to observed differences. In future studies, we aim to refine the standardization of data collection procedures to enhance the robustness and representativeness of the experimental design.

Among the identified three hub genes (AQP9, S100A9 and SLC11A1), AQP9 and S100A9 have been repeatedly implicated in inflammatory responses relevant to coronary artery disease and AMI. AQP9 is expressed in myeloid cells and has been associated with neutrophil and macrophage function as well as lipid metabolism, suggesting potential links between metabolic dysregulation and inflammation (22). Another study confirmed that AQP9 is a direct target gene of miR-212. Through inhibition of the PI3K/Akt pathway, miR-212 reduces AQP9 gene expression, thereby modulating the inflammatory response and myocardial damage (23). Previous studies have shown that AQP9 plays a critical role in lipid metabolism. In the liver, AQP9-mediated glycerol uptake is a key step in glycerol synthesis, while in adipose tissue, AQP9 is involved in lipid degradation. Mice with AQP9 gene deficiency exhibit elevated plasma triglyceride and cholesterol levels, highlighting its role in lipid homeostasis (24, 25). Given the direct link between lipid metabolism and atherosclerosis, AQP9 may contribute to atherosclerotic progression by regulating both lipid metabolism and inflammatory responses (26).

S100A9, often forming the S100A8/A9 complex, is a prototypic alarmin abundantly expressed in neutrophils and monocytes (27, 28) and can promote cytokine production through receptors such as TLR4 and RAGE (29). Studies have shown that in patients with bacterial infections, S100A9 exerts dual effects: on the one hand, it binds to calmodulin in cardiomyocytes, thereby reducing myocardial contractility; on the other hand, it induces neutrophil chemotaxis through the RAGE receptor and triggers an inflammatory cascade via the TLR/NF-*κ*B and MYD88 signaling pathways (30, 31). Building on this, another study demonstrated that S100A9 is associated with myocardial injury following AMI. The use of S100A9 inhibitors was found to regulate the expression of proteins involved in five biological processes: leukocyte adhesion, cardiomyocyte apoptosis, intrinsic apoptotic signaling, sarcomere organization, and myocardial hypertrophy. By downregulating the expression of inflammatory markers such as MOESN (32), S100A9 inhibition reduces the inflammatory response in AMI. S100A9 also plays a pivotal role in atherosclerosis development, which is recognized as a chronic inflammatory process. S100A9 promotes neutrophil adhesion to endothelial cells and enhances macrophage uptake of LDL-C, thereby accelerating the inflammatory cascade (33). A study using S100A9-targeting antibodies demonstrated a reduction in atherosclerosis-associated inflammation, suggesting that this approach could potentially serve as an alternative to lipid-lowering therapies in the future, offering a novel strategy for the clinical treatment of coronary artery disease (34).

SLC11A1 (also known as NRAMP1) emerged as a hub gene in our integrative analysis. SLC11A1 encodes a proton-coupled divalent metal ion transporter that shapes myeloid-cell iron/manganese handling and inflammatory activation (35–37). Although SLC11A1 has been implicated in macrophage/foam-cell biology in atherosclerosis and has appeared in transcriptomic biomarker signatures of coronary artery disease (38, 39), its association with AMI has not been systematically characterized. In our study, SLC11A1 was consistently upregulated in AMI and correlated with myeloid cell fractions, supporting its potential relevance to acute coronary inflammation. Mechanistically, SLC11A1 encodes a proton-coupled divalent metal ion transporter predominantly expressed in macrophages and localized to late endosomal/lysosomal membranes. By regulating iron and manganese handling within myeloid cells, SLC11A1 may influence oxidative stress, inflammatory signaling, and macrophage activation states (37). Given the importance of monocyte-to-macrophage differentiation in plaque inflammation and post-infarction repair, elevated SLC11A1 expression in AMI may reflect heightened innate immune activation and altered metal metabolism during acute coronary events. This hypothesis warrants direct experimental testing in future work, including functional assays in macrophages and *in vivo* models.

Several limitations should be noted. First, the discovery datasets were derived from PBMCs rather than myocardial tissue, and CIBERSORT estimates may not fully capture the complexity of circulating immune states. Second, our RT-qPCR validation cohort was small and lacked healthy controls; although we used an external dataset for complementary validation, larger multi-center cohorts are needed. Third, we validated mRNA expression only; protein-level validation and functional experiments were not performed. Finally, although our analyses suggest associations with immune infiltration, causality cannot be inferred from transcriptomic correlations alone.

## 5 Conclusion

In summary, we identified immune-related hub genes and molecular subtypes in AMI using an integrated bioinformatics framework and preliminary validation. AQP9, S100A9, and SLC11A1 were consistently upregulated in AMI and may serve as candidate biomarkers reflecting innate immune activation. These results provide a foundation for mechanistic studies and for developing immune-informed strategies to stratify and potentially intervene in AMI.

## Data Availability

Publicly available datasets were analyzed in this study. This data can be found here: Gene Expression Omnibus (GEO) repository (https://www.ncbi.nlm.nih.gov/geo/), accession numbers: GSE59867,GSE62646 and GSE60993.
