# Supplementary material for: Clinically validated immune-related gene markers and molecular subtypes in acute myocardial infarction revealed by peripheral blood transcriptomics
Source: Front Cardiovasc Med. 2026 Jan 23;13:1643959. doi: 10.3389/fcvm.2026.1643959 (PMC12876223; doi:10.3389/fcvm.2026.1643959)

**Table S1 GO annotation of differentially expressed genes**


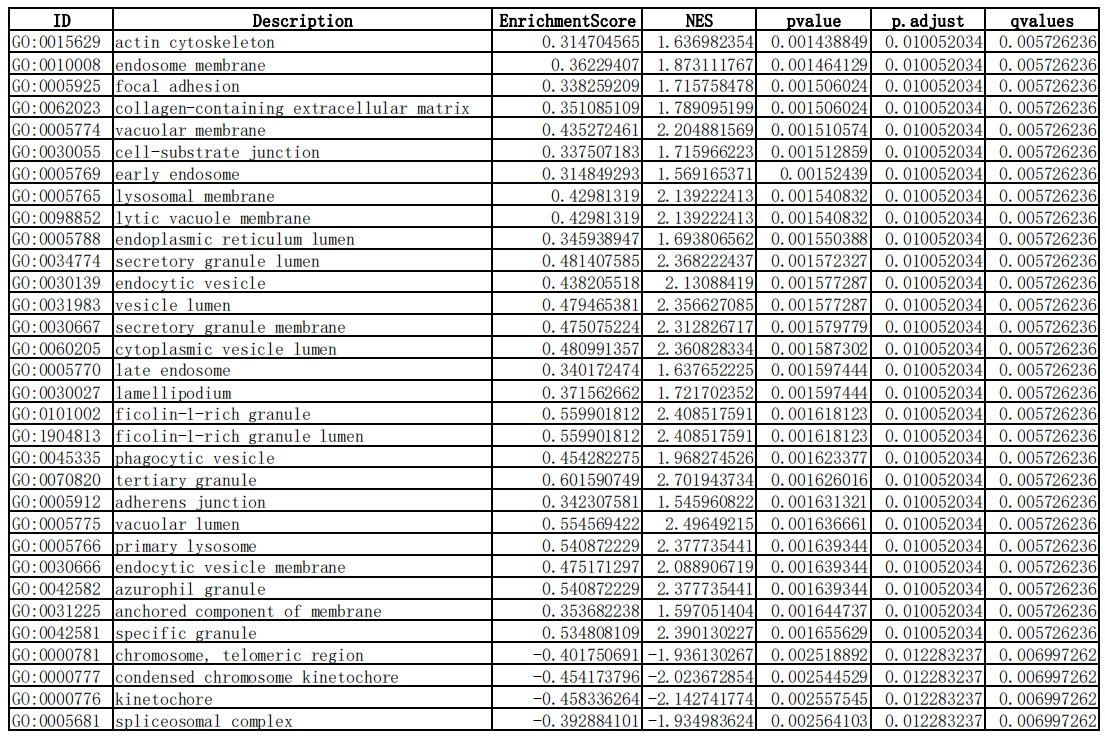


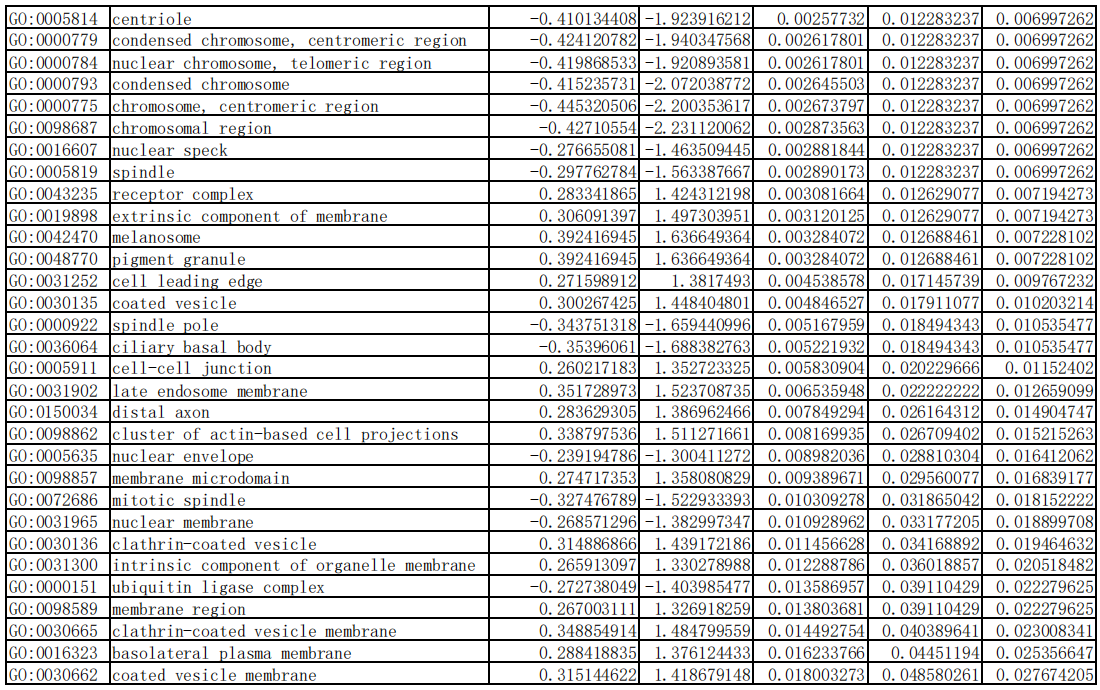


**Table S2 KEGG pathway enrichment analysis of differentially expressed genes**


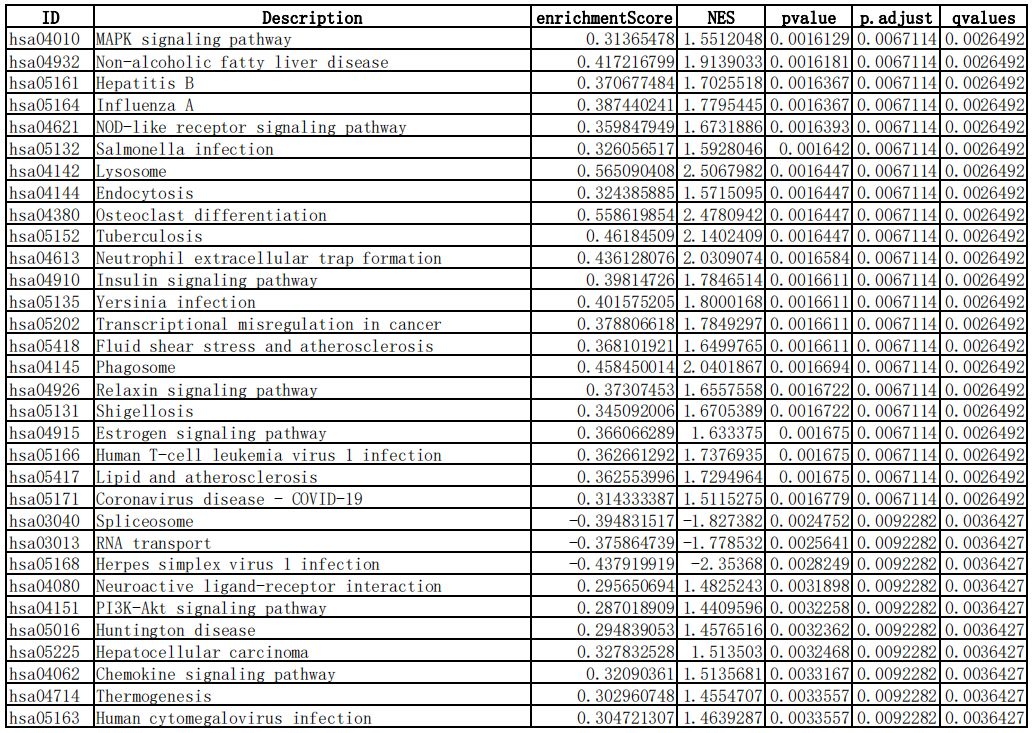


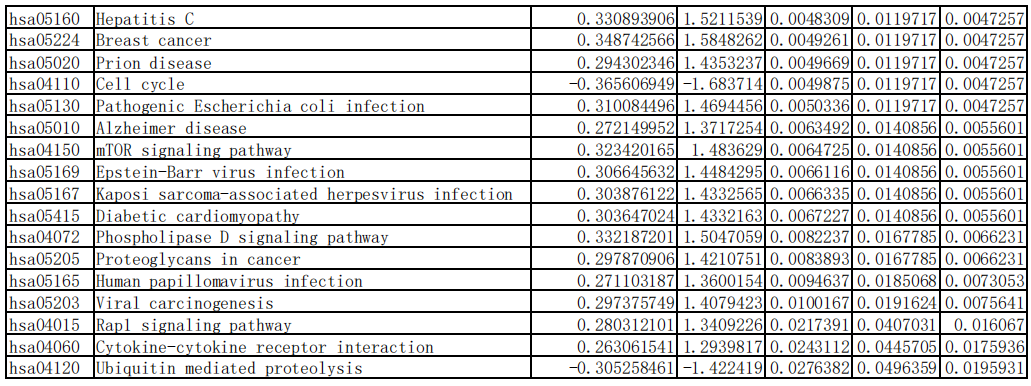


**Table S3 GO annotation of differential immune-related genes**


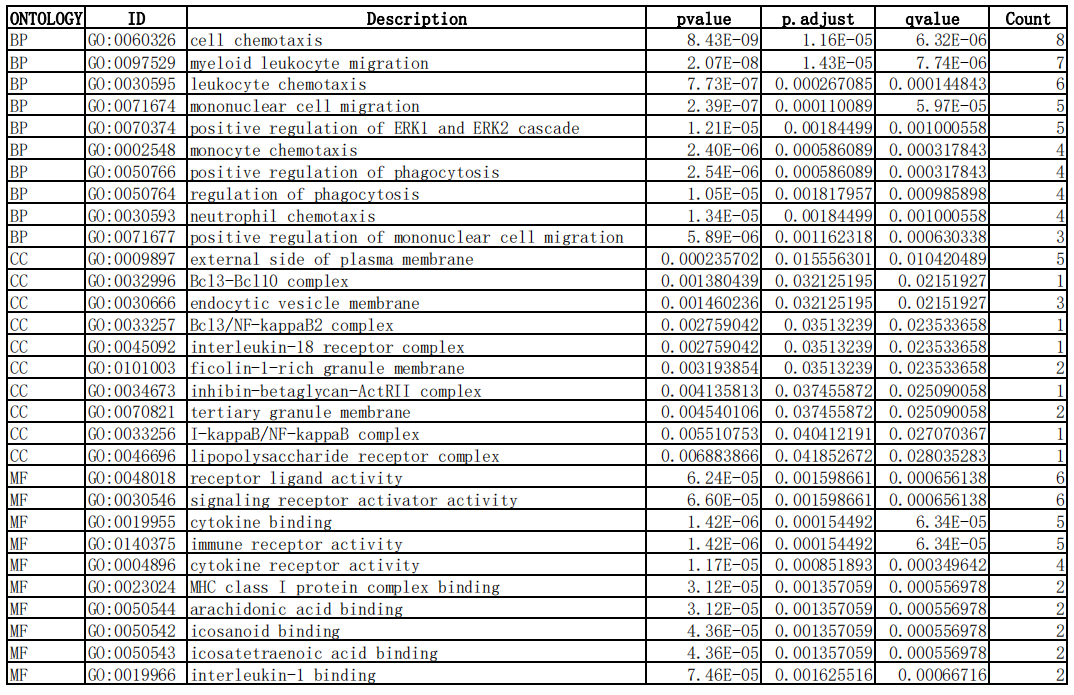


**Table S4 KEGG pathway enrichement analysis of differential immune-related genes**


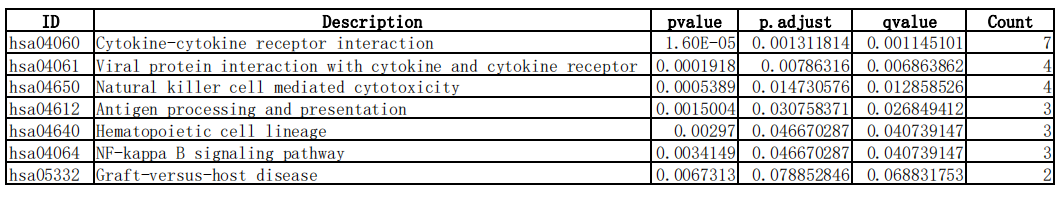

Supplement: Supplementary file 1 [file Datasheet1.docx]
