# Supplementary material for: Clinically validated immune-related gene markers and molecular subtypes in acute myocardial infarction revealed by peripheral blood transcriptomics
Source: Front Cardiovasc Med. 2026 Jan 23;13:1643959. doi: 10.3389/fcvm.2026.1643959 (PMC12876223; doi:10.3389/fcvm.2026.1643959)
Supplement: Supplementary file 2 [file Image1.pdf]

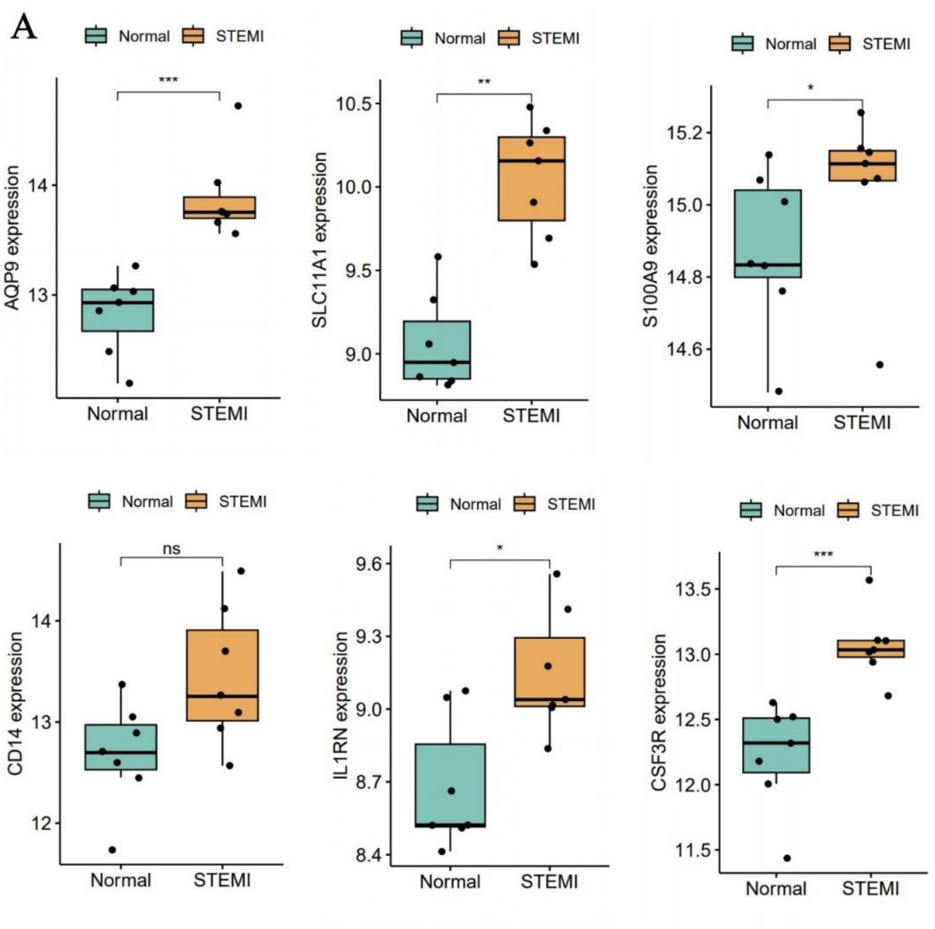

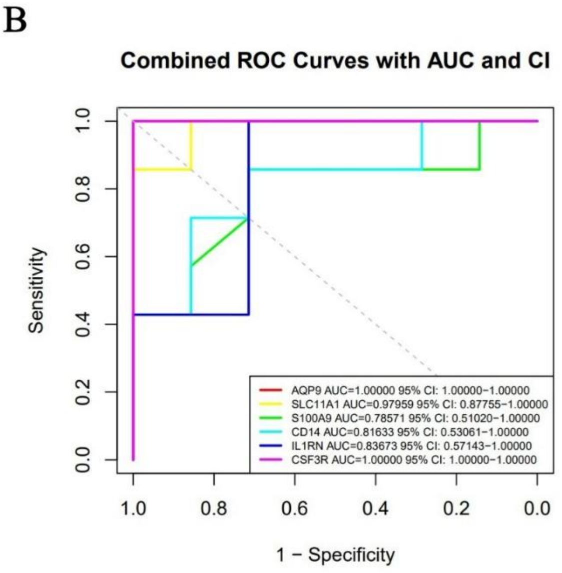

Supplementary Figure S1 Validation of hub genes inGSE60993. **(A)**Differential analysis of the six bub genes in peripheral blood. **(B)**Potential diagnostic role of the hub genes in the ROC curve in dataset.
